# Do Parents Enhance Cognitive Behavior Therapy for Youth Anxiety? An Overview of Systematic Reviews Over Time

**DOI:** 10.1007/s10567-023-00436-5

**Published:** 2023-05-22

**Authors:** S. Byrne, V. Cobham, M. Richardson, K. Imuta

**Affiliations:** 1 School of Psychology, University of Queensland, Brisbane, QLD Australia; 2 Parenting and Family Support Centre, University of Queensland, Brisbane, QLD Australia; 3 School of Psychology and Wellbeing, University of Southern Queensland, Ipswich, QLD Australia

**Keywords:** Anxiety, Youth, Review, Meta-analysis, Cognitive behavior therapy, Parent

## Abstract

The last 20 years has seen debate regarding the merits of involving parents in cognitive behavior therapy (CBT) for youth anxiety played out across systematic reviews which have high impact. These reviews examined varying treatment formats in relation to parent involvement, including youth only CBT (Y-CBT), parent only CBT (P-CBT) and family CBT (youth and parent; F-CBT). This is a novel overview of systematic reviews examining evidence for parental involvement in CBT for youth anxiety over the period this was studied. Two independent coders systematically searched for studies in medical and psychological databases using the categories “Review”, “Youth”, “Anxiety”, “Cognitive Behavior Therapy” and “Parent/Family”. Of the 2,189 unique articles identified, there were 25 systematic reviews since 2005 which compared the effects of CBT for youth anxiety with varying parent involvement. Despite systematically studying the same phenomenon, the reviews were heterogeneous in outcome, design, inclusion criteria and often had methodological limitations. Of the 25 reviews, 21 found no difference between formats and 22 reviews were considered inconclusive. Yet while there were typically no statistical differences, consistent patterns in the direction of effects were detected over time. P-CBT was less effective than other formats, suggesting the importance of directly treating anxious youths. Early reviews favored F-CBT over Y-CBT, however, later reviews did not show this trend. We consider the effects of moderators including exposure therapy, long-term outcomes and the child’s age. We consider how to manage heterogeneity in primary studies and reviews to better detect treatment differences where they exist.

*Trial registration* This protocol is registered with the Open Science Framework: osf.io/2u58t.

**Supplementary Information:**

The online version contains supplementary material available at 10.1007/s10567-023-00436-5.

Psychotherapy often assumes an important role for parents and families in the maintenance and remission of a youth’s mental disorder (e.g., Goldenberg & Goldenberg, 2012; Kazdin, 1997; Wei & Kendall, 2014). Cognitive behavior therapies (CBTs) have shown success for treating anxious young people across reviews (e.g., James et al., 2020) and overviews (e.g., Bennett et al., 2016; Manassis et al., 2010). For example, Bennett et al. (2016) examined 22 high quality reviews finding efficacy for CBT and antidepressants for child and adolescent anxiety. Attempts have been made to enhance these treatments by involving parents: parents can gain skills to manage their child's anxiety, they can learn skills to manage their own anxiety and parent involvement may promote recovery after treatment (Cobham et al., 1998; Creswell & Cartwright-Hatton, 2007; Wei & Kendall, 2014). Yet compared to other psychotherapies, CBT is more focused on the youth’s skill acquisition, so targeting them in treatment may be preferable in some cases. Youth anxiety can also be marked by child-parent negativity, enmeshment and low autonomy (e.g., Rapee et al., 2009), such that greater parent involvement could potentially detract from their recovery. As Barmish and Kendall (2005) stated “…we must resist the intuitive appeal to conclude that the inclusion of parents as active participants in CBT is preferable to child-focused CBT until the data provide the needed support….” (p. 578). Much of the debate regarding the merits of parent involvement in CBT has played out across systematic reviews which summarize and aggregate research in the field.

Whether parent involvement enhances CBT outcomes for youth anxiety is one of the most highly reviewed topics in clinical psychology. At the time of writing, there are several reviews and meta-analyses that seek to answer this question. These reviews often have high impact, they can shape the discourse in the field and influence clinical decision-making. For example, the Cochrane Review by James et al. (2020) has 1,266 citations^1^ and these reviews can influence health service delivery (e.g., National Collaborating Centre for Mental Health, 2013). Meta-analyses provide a powerful analysis by systematically merging independent studies to calculate an overall effect size. For example, Peris et al. (2021) recently conducted a meta-analysis, finding no difference between Y-CBT vs. F-CBT yet marginally favoring Y-CBT (*d* = 0.01, *p* = .86). Yet different reviews can only draw on studies available at a particular time, use set inclusion criteria and varying methodologies. For example, while some reviews compare randomized controlled trials (RCTs), others grade evidence of a treatment against set criteria or compare efficacy relative to a control. Several researchers have noted the heterogeneity of methodologies and outcomes in studies investigating parent involvement in CBT for youth anxiety (e.g., Barmish & Kendall, 2005; James et al., 2020; Thulin et al., 2014).

Different formats of CBT have also been developed in relation to the level and type of parent involvement. Alongside youth only CBT (where the youth alone attends therapy; Y-CBT) and family CBT (youth and parent attends; F-CBT), parent only CBT has begun to be integrated into reviews (P-CBT; James et al., 2020; Jewell et al., 2022). This format removes the youth from therapy and focuses on teaching parents anxiety management skills, so they can coach their child and potentially learn to manage their own anxiety (e.g., Lebowitz et al., 2020). Examining the different formats allows investigation of how parents affect anxiety outcomes, both independently and with their child. In addition, some reviews investigate how moderating variables impact parental involvement, such as the long term effects (e.g., Creswell & Cartwright-Hatton, 2007) and how parents are involved in exposure therapy (e.g., Manassis et al., 2014). There may be therapeutic and practical advantages for parent involvement in a group versus individually, so it is worth considering the moderating effects of parents across types of treatment delivery (Guo et al., 2021; Silverman et al., 2008). For example, Silverman et al. (2019) found parent-involvement in CBT reduced anxiety by reducing psychological control and group CBT reduced child anxiety by improving peer relationships. The youth’s age may also be important: younger children may benefit from greater parental involvement (e.g., Comer et al., 2019), whereas older youths may need greater autonomy (e.g., Cardy et al., 2020).

Yet a single systematic review or meta-analysis only provides a snapshot that is colored by different methodologies and outcomes of primary studies at a particular time, by contrast an overview, or a systematic review of systematic reviews, can provide a higher-level view (Fusar-Poli & Radua, 2018; Hunt et al., 2018; Pollock et al., 2016). A meta-analysis is a powerful means to aggregate data from different time-points; an overview can potentially examine patterns of effects over time and is less reliant on aggregation of data at a single time point. It can investigate directions of effects and conclusions of review authors, where consistency over time across reviews implies robustness. The aim of this study is to examine how systematic reviews report the relative effects of parent involvement on CBT for youth anxiety. We examined trends by investigating the results and conclusions of reviews over time. The dependent variable was the main quantitative measure of the youth’s anxiety (e.g., effect size of recovery rates, youth or parent reported anxiety, treatment efficacy based on level of evidence etc.). Theoretically interesting moderators identified in the course of the literature review were examined as well. To our knowledge, this is the only overview of systematic reviews examining the effects of parent involvement on youth anxiety over time.

## Method

This is an overview of systematic reviews that examines the relative efficacy of CBT for youth anxiety across Y-CBT, F-CBT and P-CBT. To provide the most comprehensive overview, we brought together systematic reviews that report a quantitative measure of youth anxiety. As the outcome of reviews was studied over time, overlap in primary studies across reviews was considered acceptable (i.e., same studies included in multiple review papers). Due to the overlapping primary studies across reviews resulting in undue influence of certain studies (particularly from earlier studies), a qualitative synthesis was conducted rather than a meta-analysis. This overview did not consider primary studies which were not part of an existing review. In order to maintain consistency, we have kept our naming convention to Y-CBT, P-CBT and F-CBT when describing similar treatments in other reviews. “Youth” will refer to both children and adolescents, however, in cases whether the sample was exclusively children or adolescents, they will be referred to as such.

The protocol of this overview is registered with the Open Science Framework—URL: osf.io/2u58t. The methodology of this overview is also described in a protocol (Byrne et al., submitted).

### Search Strategy

The search was conducted by two clinical psychologists who have experience in treating anxious youths (S.B. & M.R.). The search and final set of articles was developed in conjunction with a university librarian. The search strategy involved searching databases for articles that had four general categories: “Cognitive Behavior Therapy” AND “Youths” AND “Anxiety” AND “Review”. Of the articles identified in this broad search, the coders hand searched for articles related to “Parent/Family” treatments. The search was conducted in the electronic databases PsycINFO, Web of Science, PubMed, Cochrane Library, Scopus and EMBASE. Reference lists of selected papers were examined for additional relevant studies. The last search was conducted on July 1, 2022. See Appendix A for search queries for each database.

### Inclusion and Exclusion Criteria

#### Inclusion Criteria

1. The article must state it is a systematic review and involve a systematic search of medical and/or psychological databases.
2. The review must state the treatment used was primarily CBT.
3. The review must focus on the treatment of anxiety.
4. The review should focus on treatment for a variety of anxiety disorders (mixed anxiety; > 1 diagnoses), to reduce the potential differential effects of treatment on a particular anxiety disorder.
5. The review must report a quantitative measure (e.g., effect size, % remission etc.) related to the youth’s anxiety.
6. Parent involvement in CBT (F-CBT) could take any form (e.g., co-therapist, psychoeducation etc.), as long as treatment predominantly focused on parent and child being present and working together during therapy.
7. The review must discuss the relative efficacy of different formats for CBT. For example, it must make a direct comparison (e.g., RCTs) or an indirect comparison (e.g., compared to a control group) between the different formats of CBT for youth anxiety (Y-CBT, F-CBT or P-CBT).
8. The treatment must be for youths, so participants in reviews must be ≤ 21 years old.
9. The treatment should be primarily face-to-face psychotherapy (i.e., it should not be online or e-therapy)
10. The included reviews must be in English peer reviewed journals published from 2000 onwards.

#### Exclusion Criteria

1. Primary studies that are not systematic reviews.
2. Reviews that focused on CBT *and* another concurrent treatment for the youth’s anxiety (e.g., CBT *and* psychiatric medication). However, if a review included primary studies where some participants were receiving a concurrent treatment, they were included.
3. Reviews should not focus on comorbidity between anxiety *and* another disorder as the target of treatment (e.g., anxiety *and* autism or epilepsy); however, if a review involved primary studies where participants had comorbid conditions, they were included.
4. A review with only a narrative description of results and no comparison of treatment formats.
5. In line with the most recent Diagnostic and Statistical Manual 5^th^ Edition (American Psychiatric Association, 2013), reviews that focus on obsessive compulsive disorder (OCD) and post-traumatic stress disorder (PTSD) were excluded; however, if studies within the selected reviews included youths with these diagnoses among anxiety disorders, they were included.

### Extracted Information

The extracted data included: (1) Author name (year), (2) Overall study design, (3) Age range, (4) Analysis type between CBT formats (e.g., direct comparison, compared to control, meta-analysis etc. between Y-CBT, F-CBT and P-CBT), (5) Conclusions regarding outcome in discussion (e.g., heterogeneity), and (6) Other relevant moderators were also identified (e.g., youth age or type of treatment).

Detailed extracted data was chronologically tabled (see Table 1). The results were then summarized in a synthesized narrative over time, based on the analysis type and outcome of each review. *Cohens d* effect sizes are detailed for some comparisons in the narrative. The primary coder (S.B.) extracted the aforementioned variables for all included reviews. A second coder (M.R.) extracted the variables for 20% of included reviews (*n* = 5 reviews). The inter-rater agreement was perfect (*Cohens kappa* = 1.0).

**Table 1:** Systematic reviews comparing parent involvement in cognitive behavior therapy for youth anxiety

Author (year)	Overall study design	Age range	Analysis of CBT formats	Main results	Conclusions
Barmish and Kendall (2005)	Calculated controlled effect sizes for F-CBT and Y-CBT	6–18	Examined *N* = 9 treatment outcome studies with parent involvement. Examined effect sizes for *n* = 7 RCTs where Y-CBT or F-CBT were compared to a control	• F-CBT effect sizes comparable or larger reduction than Y-CBT for remission, youth and parent-report • Diagnostic remission for Y-CBT was medium large to large (*d* = .65-.1.20) and F-CBT was large (*d* = 1.05–2.64)	Inconclusive. High heterogeneity
James et al. (2005)	Meta-analysis of CBT for youth anxiety	6–17	Compared controlled effect sizes for Y- vs. F-CBT vs. group CBT. Analysis of recovery rate for these groups across *N* = 12 studies	• No difference in remission rate between group CBT = 56.8%; Y-CBT = 54.2%; F-CBT = 67.0% (*Chi*^*2*^ = 5.09, *p* = .07)	Inconclusive
In-Albon and Schneider (2007)	Meta-analysis of CBT for youth anxiety	6–18	Compared controlled pre-post effect sizes for Y-CBT (*n* = 16) and F-CBT (*n* = 10). Compared % of patients who recovered	• No difference for controlled pre-post overall treatment effect sizes for Y-CBT (*d* = 0.53) vs. F-CBT (*d* = 0.63; *p* = .79) • F-CBT recovery rate 76.9% vs. 64.1% Y-CBT	Inconclusive
Ishikawa et al. (2007)	Meta-analysis of CBT for youth anxiety	4–17	Reviewed *n* = 8 RCTs that directly compared Y-CBT vs. F-CBT	• F-CBT more effective than Y-CBT with very small effect (*d* = 0.03, *p* < .05)	Inconclusive
Creswell and Cartwright-Hatton (2007)	Reviewed efficacy of F-CBT for youth anxiety	6–18	Examined *n* = 9 RCTs that compared F-CBT vs. Y-CBT for youth anxiety. Also examined *n* = 10 uncontrolled F-CBT studies	• For *n* = 7 trials mean % diagnosis free F-CBT = 69.4%; Y-CBT = 55.8% • F-CBT was more effective in the long term	F-CBT probably superior to Y-CBT. Positive effects of F-CBT are maintained. High heterogeneity
Silverman et al. (2008)	Graded evidence and meta-analysis of psychosocial treatments for youth anxiety disorders	Not reported	Psychosocial treatments graded according to evidence-base based on Chambless and Hollon (1998) from Level 1 “Well-Established” to Level 4 “Experimental”. Y-CBT and F-CBT in groups or individual was compared from pre- to post-treatment vs. waitlist	• Y-CBT and group CBT graded as Level 2 “Probably Efficacious” • F-CBT is possibly efficacious • Parent involvement (68% remission) was similarly efficacious as parent non-involvement (64% remission) • Parent involvement less favourable on anxiety symptoms when rated by youth for individual (*d* = .46 vs. .31) and group formats (*d* = .41 vs. .38)	Inconclusive. Parent involvement did not significantly improve treatment outcome for individual or group treatment
Fjermestad et al. (2009)	Examined parental relationship factors on outcome of CBT with anxious youths	6–21	*N* = 12 RCTs compared parent vs. youth participation for treatment involvement and therapeutic relationship with anxiety outcome	• 6/12 RCTs found parental participation associated with change in diagnosis, symptom severity and functioning • 7 RCTs reported follow-up favoring parent participation	Inconclusive. High heterogeneity
Reynolds et al. (2012)	Meta-analysis of psychotherapy for youth anxiety	2–19	*N* = 55 studies (CBT *n* = 48). Parental involvement coded as significant (involved in majority of treatment), some involvement (involved in selected sessions), minimal (targeted) and none. Some studies involved OCD and PTSD	• No difference in effect sizes relative to a control for significant parent involvement (*d* = .63), some involvement (*d* = .65), minimal involvement (*d* = .69) or none (*d* = .57) • All effect sizes medium and significant	Inconclusive
Breinholst et al. (2012)	Comparison of RCTs with Y- CBT vs. F-CBT	6–18	Y-CBT vs. F-CBT (*N* = 11)	• 6/11 trials reported improvement/trend on F-CBT, 1 reported greater improvement on Y-CBT and 4 found no difference	Inconclusive. High heterogeneity
Thulin et al. (2014)	Meta-analysis parental involvement in CBT for youth anxiety	1–17	Meta-analysis of *N* = 17 RCTs that directly compared Y-CBT vs F-CBT	• No difference between formats • Meta-analysis showed small, non-significant effect favoring of Y-CBT. (*d* = 0.10)	Inconclusive. High heterogeneity
Manassis et al. (2014)	Compared types of parental involvement in CBT for anxious youth	6–18	Participants were Group 1 (limited parental involvement), Group 2 (active parental involvement, low contingency management or transfer of control [TC]), or Group 3 (active parental involvement, high CM or TC)	• CBT with or without parental involvement was effective • Limited parental involvement group had greater remission (Group 1: 57%) than parents with low TC and CM at post-treatment (Group 2; 50%) • Greater CM and TC had fewer anxiety diagnoses from post- to 12-month follow-up	CBT with or without parents is effective. Format of parental involvement appears key (particularly CM and TC) and is important, especially for maintenance of treatment gains. High heterogeneity
James et al. (2015)	Updated meta-analysis of CBT for youth anxiety	4–18	Compared remission vs. control and reduction in anxiety symptoms for group CBT, Y-CBT and F-CBT	• On remission rates, no difference between Y-CBT, group CBT and F-CBT (*Chi*^*2*^ = 0.06, p = .97) • Group CBT and F-CBT had greater reduction in anxiety symptoms vs. Y-CBT (*Chi*^*2*^ = 7.48, *p* = .02)	Inconclusive. High heterogeneity
Higa-McMillan et al. (2016)	Graded evidence of meta-analysis of treatment outcome studies for youth anxiety	2–19	Used 5 level system to grade treatments: Level 1 “Well Established” to Level 5 “No Support/Treatment of Questionable Efficacy” (Southam-Gerow & Prinstein, 2014). Compared within group effect sizes	• F-CBT (CBT with parents) was a Level 1 “Well Established” treatment (within group *d* = 1.25). P-CBT Level 4 “Minimal Support/Experimental Treatments” (*d* = .68)	Greater evidence for F-CBT vs. P-CBT
Öst and Ollendick (2017)	Meta-analysis of brief, intensive and concentrated CBT for youth anxiety	4–18	*N* = 23 RCTs. Parent involvement classified as low, moderate and high	• Low parent involvement (*g* = .82), moderate parental involvement (*g* = .67) and high parental involvement (*g* = -.16) • Degree of parents’ involvement inversely related to anxiety outcomes (*Q*_*b*_ = 16.1, *p* < .001)	Parents reduce efficacy of brief anxiety interventions. Parent may prevent autonomy during exposures
Zhang et al. (2017)	Meta-analysis of efficacy and acceptability of psychotherapies for young children with anxiety	2.7–9	*N* = 7 studies that compared treatment vs. control on primary efficacy outcomes. Subgroup analysis examining impact of parental involvement on effect sizes	• No difference in controlled effect sizes for F-CBT (*d* = 0.98) vs. P-CBT (*d* = 0.69; *Chi*^*2*^ = 1.23, *p* = .27)	Inconclusive
Carnes et al. (2018)	Compared F-CBT with mothers and fathers vs. Y-CBT for youth anxiety	6–17	*N* = 5 RCTs examined presence of both parents in F-CBT vs. Y-CBT at post and 1 year follow up	• No difference including both parents in F-CBT vs. Y-CBT at post and 1 year	Involving mothers and fathers did not improve outcomes. High heterogeneity
Zhou et al. (2019)	Pairwise and network meta-analysis psychotherapy for youth anxiety	≤ 18	*N* = 101 RCTs of any psychotherapy vs. another treatment/control for youth anxiety. Measured change in anxiety scores from baseline. Pairwise meta-analysis on efficacy outcomes. Also compared and ranked network using meta-analysis	• Group CBT, Y-CBT and F-CBT were more effective than waitlist at post-treatment and follow up for pairwise analysis • Only group CBT more effective than some other conditions and psychotherapies in network meta-analysis • P-CBT was non-significantly less effective than F-CBT (*d* = .14) and Y-CBT (*d* = .29). F-CBT was non-significantly less effective the Y-CBT (*d* = .14) for network meta-analysis	Inconclusive. Group CBT may be initial treatment of choice
Comer et al. (2019)	Grade evidence for treatment of anxiety and related problems in younger children	Mean = 3.9–7.7	Used 5 level system to grade treatments: Level 1 “Well Established to Level 5 “No support/Treatment of Questionable Efficacy” (Southam-Gerow & Prinstein, 2014)	• F-CBT “Well Established”. Group P-CBT and Group P-CBT + Group Y-CBT “Probably Efficacious”	Only F-CBT and Group P-CBT (with or without youth involvement) demonstrated efficacy
James et al. (2020)	Updated meta-analysis of CBT for youth anxiety	2–18	Meta-analysis of 87 RCTs of CBT vs. control	• On remission, Y-CBT superior to F-CBT and P-CBT (*Chi*^*2*^ = 8.57, *p* = .01) • Y-CBT also more effective on reduction of all anxiety disorders and youth-reported anxiety than other formats	Inconclusive. Stronger effects for Y-CBT vs. F-CBT and P-CBT. High heterogeneity
Cardy et al. (2020)	Efficacy of CBT for adolescent anxiety. Strength of evidence examined	Mean = 13.3–15.8	*N* = 23 studies were synthesized including RCTs, case studies and case series	• F-CBT for adolescent anxiety is an effective intervention • Uncertainty if parental involvement enhances treatment for adolescents	Inconclusive. High heterogeneity
Sigurvinsdóttir et al. (2020)	Meta-analysis of RCTs for CBT for youth anxiety	3–18	Compared remission for *N* = 5 RCTs that examined Y-CBT vs. F-CBT	• No significant difference between Y-CBT and F-CBT (*z* = 0.34, *p* = 0.73 favoring F-CBT)	Inconclusive
Guo et al. (2021)	Meta-analysis of individual and group CBT for youth anxiety	7–17	*N* = 9 RCTs comparing individual CBT vs. group CBT for youth anxiety, with subgroup analysis examining impact of parental involvement	• No difference group F-CBT vs. Y-CBT for individual or group treatment	Inconclusive
Peris et al. (2021)	Meta-analysis of RCTs for CBT for youth anxiety	6–18	*N* = 11 RCTs that conduct direct comparison of Y-CBT vs. F-CBT	• Across studies, no difference between Y-CBT and F-CBT for youth anxiety outcomes (*d* = 0.01, *p* = .86 favoring Y-CBT)	Inconclusive. High heterogeneity
Yin et al. (2021)	Meta-analysis of efficacy and acceptability of group P-CBT vs. group F-CBT for youth anxiety	2.7–14	*N* = 3 RCTs compared efficacy of group P-CBT vs. waitlist and F-CBT vs. waitlist	• Group F-CBT was non-significantly more effective than group P-CBT (*d* = 0.21 *p* = 0.17) • Group P-CBT was more effective than a waitlist	Inconclusive. High heterogeneity
Jewell et al. (2022)	Meta-analysis examining efficacy of P-CBT for youth anxiety	4–17	*N* = 29 studies, predominantly CBT-based. Of these *n* = 7 compared P-CBT vs. alternate treatment	• P-CBT superior to waitlist, based on parent-rated child anxiety • No significant difference between P-CBT and other formats (*n* = 7 Y-CBT; (parent rated *z* = 1.22, *p* = .22; youth-rated *z* = 1.58, *p* = .11)	Inconclusive. High heterogeneity

### Study Overlap Across Reviews

For this analysis, we only considered primary studies that were relevant to our F-CBT vs. P-CBT vs. Y-CBT analyses. A matrix table will display the extent to which there is primary study overlap across reviews. We calculated percentage overlap (proportion studies repeatedly appearing in reviews). We calculated covered area (CA) and corrected covered area (CCA) as measures of overlap.^2^ CCA classifies overlap as low (0–5%), moderate (5–10%) or high (10–15%; Pieper et al., 2014).

### Quality Assessment

Each systematic review was graded using A Measurement Tool to Assess Systematic Reviews 2nd Edition (AMSTAR 2; Shea et al., 2017). The AMSTAR 2 is a commonly used tool for assessing the methodological quality of systematic reviews and has been used to assess overviews. The AMSTAR 2 has 16 items and seven critical domains: protocol registration, adequate literature search, justification of excluding studies, risk of bias of individual studies, appropriateness of meta-analysis, consideration of risk of bias when interpreting results and assessment of publication bias. For the purposes of this study, the AMSTAR 2 rating was calculated as high (none or one non-critical weakness), moderate (more than one non-critical weakness) or low (one or more critical weaknesses). Quality ratings were completed independently by S.B. and M.R. and any disagreement was resolved through discussion.

### Results

**Fig. 1:**
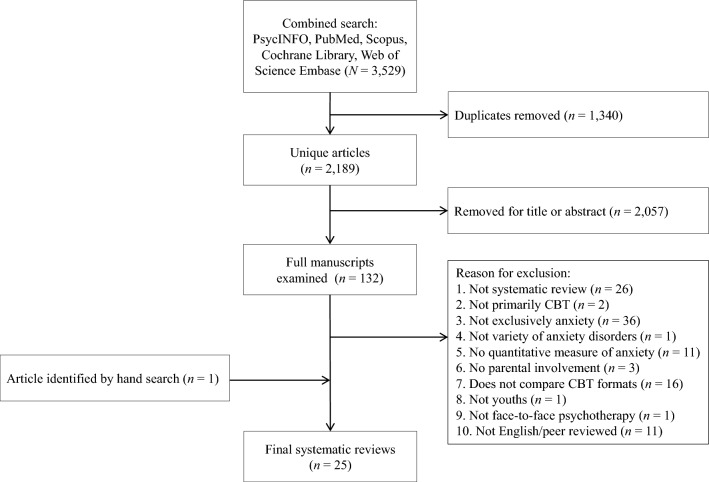
Flow chart for overview

The combined search yielded 3529 articles. Of these, 1340 were duplicates, leaving 2189 unique articles. Of these unique articles, 2057 articles were excluded on the basis of their title, abstract or non-peer reviewed article status. The full text of the final 132 articles was closely examined and 24 of these met the inclusion criteria. In addition, the authors found one study by Creswell and Cartwright-Hatton (2007) which was not identified in the search. See Fig. 1 for the PRISMA systematic review flow chart. See Table 1 for the final review papers arranged chronologically with analysis and outcome. The full references for the 25 review papers are marked with an asterisk in the Reference section. As the relevant analysis in James et al. (2013) and James et al. (2015) were the same, we have only cited the latter. The reviews were published between 2005 and 2022. The authors of the reviews were primarily based in the UK (*n* = 7), USA (*n* = 5), China (*n* = 4), Sweden (*n* = 2), Australia, Denmark, Japan, Norway, Canada, Iceland and Switzerland (all *n’s* = 1).

### Overview of the Literature

#### Overall Findings

There were 29 comparisons in anxiety outcomes between treatment formats (P-CBT vs. Y-CBT; P-CBT vs. F-CBT; Y-CBT vs. P-CBT) which were reported across 25 reviews. Of these comparisons, only five papers showed statistically significant differences of one format over another using a meta-analysis: two favoring F-CBT over Y-CBT (Ishikawa et al., 2007; James et al., 2015) and two favoring Y-CBT over F-CBT (James et al., 2020; Öst & Ollendick, 2017). One review showed P-CBT was less effective than Y-CBT (James et al., 2020). Of the 25 reviews only three made conclusions regarding the superiority of a format: Öst and Ollendick (2017) concluded that Y-CBT is superior to F-CBT when conducting brief treatments. Furthermore, Creswell and Cartwright-Hatton (2007) concluded F-CBT is probably more effective than Y-CBT. Higa-McMillan et al. (2016) concluded F-CBT has more evidence than P-CBT. Hence, 84% of reviews found no statistical difference between formats and 88% concluded no difference.

#### Methodologies

Of the 25 reviews, 18 used meta-analyses, six calculated outcomes/effect sizes for individual studies, three graded evidence according to criteria (e.g., Chambless & Hollon, 1998) and one conducted a network meta-analysis (some reviews used more than one analysis). Of the 29 comparisons, 20 studies compared Y-CBT to F-CBT, seven compared F-CBT to P-CBT and two compared Y-CBT to P-CBT (some studies made multiple comparisons). The inclusion criteria of each review varied considerably (see Online Appendix B). For example, 10 reviews stated they would include non-published articles (40%), six included subclinical samples (24%), five included non-RCTs (20%) and five included OCD (20%). The youths’ age in reviews also varied significantly between 2 to 21 years, however, two reviews focused exclusively on younger children (Comer et al., 2019; Zhang et al., 2017) and one focused on adolescents (Cardy et al., 2020). Furthermore, four reviews examined the efficacy of group versus individual anxiety CBT with varying parental involvement (Guo et al., 2021; Silverman et al., 2008; Yin et al., 2021; Zhou et al., 2019). Each review integrated between six and 121 primary studies; however, there was overlap across these papers (i.e., same studies included in multiple review papers). Of the 25 reviews, heterogeneity in primary studies was reported in the discussion in most reviews (*n* = 13 or 52%; see Table 1).

#### Study Overlap

A citation matrix is presented in Online Appendix C. Of the primary studies, 34.0% were present in more than one review. The covered area was 8.6%. The corrected covered area was 4.8%, indicating low overlap.

#### Quality

With the exception of the three Cochrane Reviews (James et al., 2005, 2015, 2020) and Zhou et al. (2019), the quality of reviews was low when assessed by the AMSTAR 2. This measure is relatively stringent, with one non-critical weakness reducing the quality grading. Of the critical domains in the AMSTAR 2, the number that fully or partially fulfilled each criteria was: protocol registered (*n* = 7; 28%), adequacy of literature search (*n* = 22; 88%), justification for excluding individual studies (*n* = 4; 16%), risk of bias (*n* = 13; 52%), appropriateness of meta-analytic techniques (*n* = 19/19; 100%), risk of bias when interpreting results (*n* = 13; 52%) and assessment of publication bias (*n* = 13/19; 68.4%).

### Summary of Findings

#### Parent Only Cognitive Behavior Therapy versus Other Formats

The reviews that examined P-CBT were fewer in number and more recent than for other comparisons. A single Cochrane Review by James et al. (2020) found that P-CBT was associated with statistically lower remission, lower remission of all anxiety disorders and greater child-reported anxiety than Y-CBT, yet was comparable to F-CBT. The authors, however, concluded the high heterogeneity prevented one format being considered superior to the others. All other meta-analyses reported non-significant findings, however, their direction of effects consistently favored other formats over P-CBT. Zhang et al. (2017) found no difference between F-CBT versus P-CBT on primary efficacy outcomes relative to a control, yet effects slightly favored F-CBT (*d* = 0.98 vs. 0.69). Zhou et al. (2019) conducted a meta-analysis of psychotherapies for anxiety, finding group CBT, Y-CBT and CBT with parental involvement were more effective than a waitlist on anxiety symptoms. They also found no difference between any format for a network meta-analysis, except group CBT was often more effective than some other formats. Yin et al. (2021) synthesized six RCTs examining the efficacy of group P-CBT versus F-CBT or a waitlist, finding group P-CBT was more effective than waitlist, yet not different to F-CBT (*d* = 0.21 favoring F-CBT). A review by Jewell et al. (2022) reviewed 29 studies which examined parent-only interventions employing RCTs and case series designs. They found P-CBT was superior compared to a waitlist, but not compared to active interventions (mainly Y-CBT) on parent and youth rated anxiety outcomes. The authors noted the methodological quality of the primary studies was generally weak.

In addition to conducting a meta-analysis, some studies have graded P-CBT compared to the other formats using set criteria (Southam-Gerow & Prinstein, 2014). For example, according to these criteria, a “Well-Established Treatment” can show efficacy in two experiments conducted in independent settings. Higa-McMillan et al. (2016) categorized “treatment families”—that is, treatment approaches with similar clinical strategies and theory. They found P-CBT (“Minimal Support/Experimental”; one RCT showing treatment superior to a waitlist) was less effective than F-CBT (“Well Established”; *d* = 0.68 versus *d* = 1.25 for within group effect sizes). Comer et al. (2019) later examined family-based treatments for children with a mean age under 7.9 years, describing variations of Group P-CBT as “Probably Efficacious” compared to F-CBT which was “Well Established”. Collectively, these results suggest P-CBT is effective, however, it may be less effective than other formats.

#### Family Versus Youth Cognitive Behavior Therapy

The F-CBT versus Y-CBT literature is more numerous and outcomes are more heterogeneous than for P-CBT. Similar to the literature on P-CBT, the majority of the reviews studying F-CBT versus Y-CBT have not reported statistically significant findings. In terms of direction of effects, however, there appears to be a shift across time: earlier reviews provided some evidence that favored F-CBT, whereas later reviews showed no difference or a slight advantage to Y-CBT. An early review by Barmish and Kendall (2005) compared effect sizes for Y-CBT and F-CBT versus a control across eight RCTs. They reported larger controlled effect sizes for F-CBT for some studies and on some indices (e.g., Barrett, 1998; Barrett et al., 1996). For example, the controlled effect sizes for remission were typically larger for F-CBT (*d* = 1.05–2.64) than for Y-CBT (*d* = 0.65–1.20). The authors concluded, however, that there was insufficient evidence for differences between formats due to the variability between treatments. In the same year, a Cochrane Review by James et al. (2005) conducted a meta-analysis of remission rates across CBT formats, finding no difference yet a trend towards greater remission of F-CBT (group treatment = 56.8%; Y-CBT = 54.2%; F-CBT = 67.0%; *p* < .07). A few years later, Creswell and Cartwright-Hatton (2007) conducted a review of F-CBT RCTs and uncontrolled studies, concluding F-CBT was probably superior to Y-CBT and more effective in the long term. Around the same time, Ishikawa et al. (2007) published a meta-analysis of eight RCTs which directly compared Y-CBT versus F-CBT, finding a very small but statistically significant advantage for F-CBT on anxiety outcomes (*d* = 0.03, *p* < .05). The authors, however, described the outcome as heterogeneous and inconclusive.

Later studies were inconclusive, yet provided some evidence favoring F-CBT. In another review published in 2007, a meta-analysis by In-Albon and Schneider (2007) reported F-CBT had a slightly higher recovery rate than Y-CBT (76.9% vs. 64.1%), yet they found no difference between Y-CBT and F-CBT versus a control from pre to post in remission from diagnosis (*d* = 0.53 and 0.63 respectively). Fjermestad et al. (2009) examined parent–child relationship factors on youth anxiety treatments (RCTs and aged ≤ 21 years). While acknowledging inconsistencies within and across methodologies, half of the RCTs reviewed (6/12) found greater parent participation had a positive effect or a trend on at least one anxiety-related measure (e.g., on diagnostic status, symptom level or functioning). Similarly, Breinholst et al. (2012) reported that more than half of the reported studies found a trend or superior efficacy of F-CBT on at least one outcome, yet concluded that the outcomes were “ambiguous and inconsistent” (p. 416). Carnes et al. (2019) found the addition of both parents did not improve F-CBT vs. Y-CBT. Finally, a follow-up review by James et al. (2015) found greater reduction in anxiety symptoms for group CBT and F-CBT compared to Y-CBT, however, no difference in remission rates.

Two earlier reviews which used a graded evidence approach to compare the relative efficacy of F-CBT versus Y-CBT also support this view. Silverman et al. (2008) used criteria developed by Chambless and Hollon (1998) for empirically supported treatments to grade F-CBT versus Y-CBT. Silverman et al. (2008) described Y-CBT as “Probably Efficacious” (e.g., at least two experiments showing superiority to a waitlist or control) and F-CBT treatments as "Possibly Efficacious” (e.g., at least one good experiment showing it is efficacious). They concluded that parental involvement in individual or group CBT was as effective as non-involvement. Using a similar criteria by Southam-Gerow and Prinstein (2014), Higa-McMillan et al. (2016) categorized “treatment families”—that is, treatment approaches with similar clinical strategies. Both CBT and F-CBT were Level 1 “Well Established” treatments, reflecting the growing evidence base for both formats since Silverman et al. (2008).

Later reviews which included more primary studies typically found no statistical difference between Y-CBT and F-CBT, yet some have reported very small, non-significant effects favoring Y-CBT. Reynolds et al. (2012) conducted a meta-analysis of 55 studies which were almost exclusively CBT for youth anxiety and included OCD and PTSD. They found no difference in outcomes relative to a control for significant parent involvement (parents involved in all sessions; *d* = 0.63), for some involvement (involved in some sessions; *d* = 0.65), minimal involvement (involved in targeted sessions e.g., psychoeducation; *d* = 0.69) or no involvement (*d* = 0.57). Two reviews reported very small and non-significant effects favoring Y-CBT. Thulin et al. (2014) synthesized 16 studies where there was a comparison of F-CBT versus Y-CBT including OCD, PTSD and panic disorder. This was the first study to report a very small non-significant advantage for Y-CBT (*d* = 0.1). Similarly, Peris et al. (2021) found no difference on anxiety outcomes for Y-CBT vs. F-CBT across 11 RCTs comparing F-CBT and Y-CBT on anxiety measures (*d* = 0.01 favoring Y-CBT). Zhou et al. (2019) found both Y-CBT and F-CBT were more effective than a waitlist for pairwise comparisons. When they rank ordered different treatments, they found group CBT superior to the other formats, yet no significant difference between other CBT formats. Sigurvinsdóttir et al. (2020) examined five RCTs which directly compared Y-CBT vs. F-CBT, finding no difference in efficacy.

Finally, in one of the more recent and rigorous systematic reviews, James et al. (2020) found evidence favoring Y-CBT. They found no difference directly comparing P-CBT and F-CBT compared to a waitlist, however, both formats were less effective than Y-CBT based on remission of primary anxiety disorder, remission of all anxiety disorders and youth-reported anxiety symptoms. The authors noted the high levels of heterogeneity and therefore argue that the results were inconclusive.

### Moderators of Parent Involvement on Youth Anxiety Outcomes

#### Exposure Therapy

Exposure therapy is a fundamental component of treatment for youth anxiety and some reviews suggest parents have a variable effect on this process. Öst and Ollendick (2017) were the first to find statistical evidence that parent involvement had an inverse effect on outcomes during brief, concentrated anxiety interventions. This was possibly due to parents acting as a safety signal and reducing the youth’s self-efficacy during exposure therapy (Ollendick et al., 2015). This important result suggests for brief exposure therapy, parents can have a variable effect and may be unhelpful. Manassis et al. (2014) found both anxiety treatments with and without parent involvement were effective, however, F-CBT focusing on exposure techniques (contingency management and transfer of control) were most likely to be effective and have ongoing gains.

#### Youth Age

Most reviews used a wide age range including children and adolescents, making it difficult to delineate the relative effects of treatment formats based on youth age. A comparison of the reviews that focus on specific age groups provides some limited evidence younger children may benefit from having parents present with them. Cardy et al. (2020) identified 23 primary studies where parents were involved in adolescent anxiety treatment (mean age = 13.3–15.8 years), finding parent involvement was effective, however, they were uncertain whether it enhanced outcomes. In a review that focused on younger children (mean age < 7.9 years), Comer et al. (2019) found F-CBT was “Well Established”, whereas Group Parent CBT and Group Parent and Child CBT were “Probably Efficacious”. Amongst young children (mean age less than seven years), Zhang et al. (2017) found no difference in F-CBT and P-CBT, yet effects slightly favored F-CBT.

#### Individual versus Group Treatment

A small number of studies examined parent involvement for individual or group treatments generally finding no clear differences between formats. Using criteria developed by Chambless and Hollon (1998), Silverman et al. (2008) reported Group Y-CBT (group therapy with youths) and Group F-CBT (group therapy with youths and parents) were “Probably Efficacious”, compared to Y-CBT (individual therapy with youths) which was also “Probably Efficacious” (all formats received the same evidence grading). Later research by Comer et al. (2019) used criteria by Southam-Gerow and Prinstein (2014) finding Group P-CBT and Group F-CBT were “Probably Efficacious”, compared to F-CBT which was “Well Established” for early childhood, suggesting a possible advantage of individualized family-based treatment for younger children. Zhou et al. (2019) reported that Group CBT, Y-CBT and F-CBT were all more effective than a waitlist in a pairwise meta-analysis. Their network meta-analysis found no statistically significant differences between treatments, except that group CBT was generally more effective. Yin et al. (2021) found no statistical difference between Group P-CBT and F-CBT. Guo et al. (2021) also found no difference for parental involvement for individual or group CBT, however, they found Y-CBT was more effective for adolescents.

#### Longer Term Effects

There is some evidence parent involvement can improve longer term outcomes. Creswell and Cartwright-Hatton (2007) reported that F-CBT was more effective than Y-CBT in the longer term. Fjermestad et al. (2009) reported that studies found that parent involvement was positively associated with better long term follow-up outcomes. Manassis et al. (2014) found when parents received coaching contingency management and transfer of control was associated with better long term outcomes. Jewell et al. (2022) found that parent-only interventions may have a positive long-term effect on outcomes. Yet Carnes et al. (2019) found no difference involving both parents in treatment at 1 year follow-up.

## Discussion

The last 20 years has seen debate regarding the merits of including parents in CBT for youth anxiety which has played out across several systematic reviews. Over this period, few individual systematic reviews have shown differences between treatment formats and even when there were statistical differences, heterogeneity often left reviews as inconclusive. The systematic reviews used highly variable inclusion criteria (see Online Appendix B). With the exception of the Cochrane Reviews (e.g., James et al., 2020) and Zhou et al. (2019), the quality of these reviews was lower, which could partly explain the variation in outcomes. Nevertheless, despite the non-significant results, there was subtle variation in the direction of effects. For example, different authors arrived at different conclusions at similar times (James et al., 2020; Sigurvinsdóttir et al., 2020) and the same authors arrived at different conclusions over time (e.g., James et al., 2005, 2015, 2020). While individual reviews did not detect differences, it is possible to see consistent patterns in the direction of effects over time.

Results consistently indicate that P-CBT was less favorable than other formats. P-CBT was less effective than Y-CBT on remission and anxiety symptoms in a recent review (James et al., 2020) and every other comparison found P-CBT was non-significantly less favorable than Y-CBT and F-CBT. These results suggest that while parents can act as an intermediary between the therapist and anxious youth, it is preferable for the therapist to work directly with the youth. Nevertheless, parent-only treatment may be desirable when vicariously treating youth who are disengaged or for coaching parents in CBT skills that can be used in an ongoing way (e.g., Jewell et al., 2022; Lebowitz et al., 2020; Phillips & Mychailyszyn, 2021).

The literature comparing F-CBT versus Y-CBT is more abundant and heterogeneous than for P-CBT. There has sometimes been an assumption that including parents with their child in CBT is preferable (e.g., Barmish & Kendall, 2005), however, the findings suggest the relative efficacy of F-CBT versus Y-CBT has gradually shifted over time. Early reviews trend towards the superiority of F-CBT (e.g., Creswell & Cartwright-Hatton, 2007; Ishikawa et al., 2007), later reviews suggest no difference (e.g., Reynolds et al., 2012; Thulin et al., 2014) and a more recent large review trends towards the superiority of Y-CBT (James et al., 2020). One possible explanation for the shift in findings may be the growth in the literature over time. Early reviews were influenced by a small number of primary studies favoring F-CBT (e.g., Barrett, 1998; Barrett et al., 1996), then moved away from F-CBT, particularly with the publication of several studies in 2008 (Bodden et al., 2008; Kendall et al., 2008; Silverman et al., 2008). The more recent reviews have the greatest sample to detect differences, with a large and rigorous study by James et al. (2020) finding a trend that Y-CBT is more effective. This trend is consistent with the previous P-CBT analysis, suggesting the importance of directly treating youths versus training their parents. While speculative, this shift could also reflect changes in the family unit and dynamics over the period studied.

The reduced efficacy of F-CBT could be due to parents moderating the effects of parent-guided exposure therapy, which is a crucial component of CBT. In one of the only definitive results, parents were shown to reduce the efficacy of brief anxiety interventions, which often involved exposure therapy (Öst & Ollendick, 2017). They suggest that in these scenarios, parents may act as a safety signal, preventing the youth from developing self-efficacy. In another review, Manassis et al. (2014) found no difference between formats, however, parents trained in contingency management and transfer of control were more likely to have better outcomes. Collectively, these results suggest parents can be counterproductive during exposures and may need to master specific skills for the youth to benefit. This suggests the therapist should closely observe how parent and youth interact during exposures—for example, high emotion, clinging or conflict between the dyad may suggest a parent’s presence is unhelpful.

This overview provides some limited evidence that parent involvement may be more beneficial for younger children who are typically less cognitively developed (e.g., Comer et al., 2019). These results provide evidence that parental involvement improves longer term outcomes (e.g., Creswell & Cartwright-Hatton, 2007), possibly because parents are able to act as a surrogate therapist after formal treatment has ended. The equivalence in efficacy between treatment formats for individual and group CBT suggests other factors like acceptability and cost-effectiveness should also be considered. For example, there is evidence that group treatments may be less expensive, just as effective and more acceptable to certain clients (Aguilera-Martín et al., 2022).

The lack of difference between formats across in individual reviews is reassuring, as it suggests all treatment formats are effective to some extent (there is a “ceiling effect”). In addition, the high heterogeneity among primary studies has often meant few firm statistical conclusions can be drawn. Clinical research can be difficult to control and a meta-analysis may be less sensitive for detecting differences (Gagnier et al., 2012). Of the individual reviews included in this overview, many state heterogeneity made it harder to detect effects, so greater experimental control, delineation between formats, standardization and the use of moderators could improve sensitivity. For example, studies which directly compare treatment formats, employ randomization and control for variables like age are preferable. These results also suggest improving methodological quality of primary studies and reviews should reduce heterogeneity. For example, our results suggest establishing pre-registered protocols, managing risk of bias in individual studies and publication bias should reduce heterogeneity. Finally, as always, the discerning reader should remember to interpret the results and conclusions of systematic reviews within their methodological parameters.

An overview over time provides a high-level analysis, however, it should be interpreted in light of its limitations. A consistent pattern in effects is informative, however, it does not equate to statistical significance required for strong conclusions. Furthermore, there are a limited number of studies that meet the inclusion criteria of this overview. A general limitation is that overviews attempt to summarize summaries, such that nuance in the data could be lost, especially when observing results of heterogeneous studies. Related to this, primary studies may be left out of reviews, particularly if they are more recent (e.g., Silverman et al., 2021). It should be considered that reviews published at similar times will draw on many of the same studies (there is overlap), which partially explains the similar outcomes of reviews published at similar times, however, this overview generally showed low levels of overlap. This overview focused on youth anxiety outcomes as the dependent variable, however, change in important variables, like family dynamics and impact on functioning should also be considered (e.g., Kreuze et al., 2018). Finally, future overviews could be strengthened with further steps, such as reporting heterogeneity and potential biases, stratification of evidence and sensitivity of analysis (Fusar-Poli & Radua, 2018).

While the efficacy of CBT for youth anxiety has been established, its optimal implementation is contested. While systematic reviews are considered a gold standard for answering these questions, their conclusions are sometimes subjective: differences in outcomes reflect differences in evidence and differences in methodologies and conclusions of individual reviews. Reviews in this area typically have not detected statistical differences, often due to high heterogeneity in primary studies. Nevertheless, it is possible to detect meaningful patterns and consistency in the results over time. These results suggest the importance of prioritizing and refining direct treatment of anxious youth over and above working with their parents. Results indicate a gradual movement away from F-CBT to Y-CBT. A possible reason for this shift is change due to variation in parent-assisted exposure therapy. To our knowledge, this is the first overview to examine the relative efficacy of different CBT formats for youth anxiety using an overview methodology. While this study focused on the results and conclusions of reviews, future research should examine subtle changes in methodologies and outcomes of primary studies for F-CBT versus Y-CBT over time. With the increasing use of online treatments, future research could examine how the absence of a face-to-face therapist moderates outcomes across formats. Furthermore, examining the moderating effects of parental involvement for particular anxiety disorders and reporting type (e.g., parent, child and therapist) will be valuable. As always, research should focus on how to best tailor parent involvement to the individual youth and their family. These results suggest the importance of continuing to meaningfully synthesize, report and interpret youth anxiety outcomes as they evolve.

## Supplementary Information

Below is the link to the electronic supplementary material.

Supplementary file1 (DOCX 33 KB)

Supplementary file2 (DOCX 23 KB)

Supplementary file3 (DOCX 43 KB)

Supplementary file4 (XLSX 40 KB)

## Footnotes

1 Google Scholar, 10 July 2022.

2 CA = number of publication inclusions ÷ (number of publications × number of reviews); CCA = (number of publication inclusions – number of publications) ÷ [(number of publications × number of reviews) – number of publications].
