# Supplementary material for: Do Parents Enhance Cognitive Behavior Therapy for Youth Anxiety? An Overview of Systematic Reviews Over Time
Source: Clin Child Fam Psychol Rev. 2023 May 22;26(3):773–88. doi: 10.1007/s10567-023-00436-5 (PMC10465628; doi:10.1007/s10567-023-00436-5)
Supplement: Supplementary file 2 — Supplementary file2 (DOCX 23 KB) [file 10567_2023_436_MOESM2_ESM.docx]

**Appendix B.**

*Search queries for systematic review*

**PubMed Search**

**"cognitive behaviour therapy"[All Fields] OR "cognitive behavioral therapy"[MeSH Terms] OR ("cognitive"[All Fields] AND "behavioral"[All Fields] AND "therapy"[All Fields]) OR "cognitive behavioral therapy"[All Fields] OR ("cognitive"[All Fields] AND "behavior"[All Fields] AND "therapy"[All Fields]) OR "cognitive behavior therapy"[All Fields] OR "CBT"[All Fields] OR "cognitive behavioral therapy"[MeSH Terms] OR ("cognitive"[All Fields] AND "behavioral"[All Fields] AND "therapy"[All Fields]) OR "cognitive behavioral therapy"[All Fields] OR ("cognitive"[All Fields] AND "therapy"[All Fields]) OR "cognitive therapy"[All Fields] OR "behaviour therapy"[All Fields] OR "behavior therapy"[MeSH Terms] OR ("behavior"[All Fields] AND "therapy"[All Fields]) OR "behavior therapy"[All Fields]**

**AND**

**"minors"[MeSH Terms] OR "minors"[All Fields] OR "minor"[All Fields] OR "boy"[All Fields] OR "boys"[All Fields] OR "boyhood"[All Fields] OR "girl*"[All Fields] OR "kid"[All Fields] OR "kids"[All Fields] OR "child"[MeSH Terms] OR "child"[All Fields] OR "children"[All Fields] OR "child s"[All Fields] OR "children s"[All Fields] OR "childrens"[All Fields] OR "childs"[All Fields] OR "child*"[All Fields] OR "children*"[All Fields] OR "schoolchild*"[All Fields] OR "schoolchild"[All Fields] OR "school child"[Title/Abstract] OR "school child*"[Title/Abstract]** OR "adolescent"[MeSH Terms] **OR "adolescen*"[All Fields] OR "juvenil*"[All Fields] OR "youth*"[All Fields] OR "teen*"[All Fields] OR "under age*"[All Fields] OR "pubescen*"[All Fields] OR "pediatrics"[MeSH Terms] OR "pediatric*"[All Fields] OR "paediatric*"[All Fields] OR "peadiatric*"[All Fields] OR "school"[Title/Abstract] OR "school*"[Title/Abstract]**

**AND**

**"anxiety"[MeSH Terms] OR "anxiety"[All Fields] OR "anxieties"[All Fields] OR "anxiety s"[All Fields] OR "anxious"[All Fields] OR "anxiety disorders"[MeSH Terms] OR ("anxiety"[All Fields] AND "disorders"[All Fields]) OR "anxiety disorders"[All Fields] OR ("anxiety"[All Fields] AND "disorder"[All Fields]) OR "anxiety disorder"[All Fields] OR "phobic disorders"[MeSH Terms] OR "phobic"[All Fields] AND "disorders"[All Fields] OR "phobic disorders"[All Fields] OR "phobia"[All Fields] OR "phobias"[All Fields] OR "phobi*"[All Fields] OR "panic"[MeSH Terms] OR "panic"[All Fields] OR "panics"[All Fields] OR "panic disorder"[MeSH Terms] OR ("panic"[All Fields] AND "disorder"[All Fields]) OR "panic disorder"[All Fields] OR ("panic"[All Fields] AND "attack"[All Fields]) OR "panic attack"[All Fields] OR ("agoraphobia"[MeSH Terms] OR "agoraphobia"[All Fields] OR "agoraphobias"[All Fields]) OR "mutism"[MeSH Terms] OR "mutism"[All Fields] OR ("selective"[All Fields] AND "mutism"[All Fields]) OR "selective mutism"[All Fields] OR "selective mutism"[All Fields] OR "fear"[MeSH Terms] OR "fear"[All Fields] OR "worried"[All Fields] OR "worries"[All Fields] OR "worry"[All Fields] OR "worrying"[All Fields]**

**AND**

**(meta-analysis[Title/Abstract] OR "systematic review"[Title/Abstract] OR systematic [sb]) OR (review*[Title/Abstract] AND ("randomized controlled trial*"[Title/Abstract] OR "randomised controlled trial*"[Title/Abstract] OR RCTs[Title/Abstract] OR RCT[Title/Abstract]))**

**Web of Science**

**TS=("cognitive behavio?r* therapy" OR cbt OR "cognitive therapy" OR "behavio?r* therapy")**

**AND**

**TS=(child OR child* OR minor OR minors OR boy* OR girl* OR schoolchild* OR adolescen* OR teen* OR youth* OR pubescen* OR p?ediatric* OR juvenil* )**

**AND**

**TS=( anxiet* OR phobi* OR panic OR agoraphobi* OR "selective mutism" OR worry OR fear)**

**AND**

**TS=(meta-analysis OR "systematic review" OR (review* AND ("randomized controlled trial*" OR "randomised controlled trial*" OR RCT*)))**

Scopus

(( ( TITLE-ABS-KEY ( "cognitive behavio?r* therapy" ) ) OR ( TITLE-ABS-KEY ( cbt ) ) OR ( TITLE-ABS-KEY ( "cognitive therapy" ) ) )) OR (TITLE-ABS-KEY(behavio?r therapy))

AND

(( ( TITLE-ABS-KEY ( child ) ) OR ( TITLE-ABS-KEY ( child* ) ) OR ( TITLE-ABS-KEY ( minor ) ) OR ( TITLE-ABS-KEY ( minors ) ) OR ( TITLE-ABS-KEY ( boy ) ) OR ( TITLE-ABS-KEY ( girl ) ) OR ( TITLE-ABS-KEY ( schoolchild ) ) OR ( TITLE-ABS-KEY ( adolescen* ) ) OR ( TITLE-ABS-KEY ( teen* ) ) OR ( TITLE-ABS-KEY ( youth ) ) OR ( TITLE-ABS-KEY ( pubescen* ) ) OR ( TITLE-ABS-KEY ( p?ediatric* ) ) )) or (TITLE-ABS-KEY(juvenil*))

AND

(( ( TITLE-ABS-KEY ( anxiety ) ) OR ( TITLE-ABS-KEY ( phobi* ) ) OR ( TITLE-ABS-KEY ( panic ) ) OR ( TITLE-ABS-KEY ( agoraphobia ) ) OR ( TITLE-ABS-KEY ( "selective mutism" ) ) )) OR (TITLE-ABS-KEY(worry OR fear))

AND

( ( TITLE-ABS-KEY ( meta-analysis ) ) OR ( TITLE-ABS-KEY ( "systematic review" ) ) OR ( TITLE-ABS-KEY ( review* AND ( "randomized controlled trial*" OR "randomised controlled trial*" OR RCT* ) ) ) )

Cochrane

ID Search Hits

#1 MeSH descriptor: [Cognitive Behavioral Therapy] explode all trees

#2 ("cognitive behaviour therapy"):ti,ab,kw (Word variations have been searched)

#3 (cognitive AND behavio?r* AND therapy):ti,ab,kw (Word variations have been searched)

#5 ("cognitive behavioral therapy"):ti,ab,kw (Word variations have been searched)

#6 (CBT):ti,ab,kw (Word variations have been searched)

#7 MeSH descriptor: [Behavior Therapy] explode all trees

#8 (behavior therapy):ti,ab,kw (Word variations have been searched)

#9 (behavio?r AND therapy):ti,ab,kw (Word variations have been searched)

#10 ("cognitive therapy"):ti,ab,kw (Word variations have been searched)

#11 (cognitive AND therapy):ti,ab,kw (Word variations have been searched)

#12 {OR #1-#11}

#13 MeSH descriptor: [Minors] explode all trees

#14 MeSH descriptor: [Child] explode all trees

#15 MeSH descriptor: [Pediatrics] explode all trees

#16 ("minors" OR "minor" OR "boy" OR "boys" OR "boyhood" OR "girl*" OR "kid" OR "kids" OR "child" OR "children" OR "child s" OR "children s" OR "childrens" OR "childs" OR "child*" OR "children*" OR "schoolchild*" OR "schoolchild" OR "adolescen*" OR "juvenil*" OR "youth*" OR "teen*" OR "under age*" OR "pubescen*" OR "pediatric*" OR "paediatric*" OR "peadiatric*"):ti,ab,kw (Word variations have been searched) 199019

#17 ("school child" OR "school child*" OR "school" OR "school*"):ti,ab,kw (Word variations have been searched)

#18 {OR #13-#17}

#19 MeSH descriptor: [Anxiety] explode all trees

#20 MeSH descriptor: [Anxiety Disorders] explode all trees

#21 MeSH descriptor: [Phobic Disorders] explode all trees

#22 MeSH descriptor: [Panic] explode all trees

#23 MeSH descriptor: [Panic Disorder] explode all trees

#24 MeSH descriptor: [Agoraphobia] explode all trees

#25 MeSH descriptor: [Mutism] explode all trees

#26 MeSH descriptor: [Fear] explode all trees

#27 ("anxiety" OR "anxieties" OR "anxiety s" OR "anxious" OR ("anxiety" AND "disorders" ) OR "anxiety disorders" OR ("anxiety" AND "disorder" ) OR "anxiety disorder" OR "phobic" AND "disorders" OR "phobic disorders" OR "phobia" OR "phobias" OR "phobi*" OR "panic" OR "panics" OR ("panic" AND "disorder" ) OR "panic disorder" OR ("panic" AND "attack" ) OR "panic attack" OR "agoraphobia" OR "agoraphobias" OR "mutism" OR ("selective" AND "mutism" ) OR "selective mutism" OR "selective mutism" OR "fear" OR "worried" OR "worries" OR "worry" OR "worrying"):ti,ab,kw (Word variations have been searched)

#28 {OR #19-#27}

#29 #12 AND #18 AND #28

#30 ((meta-analysis OR "systematic review") OR (review* AND ("randomized controlled trials" OR "randomised controlled trials" ))):ti,ab,kw (Word variations have been searched)

#31 #29 AND #30

PsycINFO

((MeSH: (cognitive behavioral therapy)) OR (Any Field: ("cognitive behavio?r* therapy")) OR (Any Field: (CBT)) OR (Any Field: ("cognitive therapy")) OR (Any Field: ("behavio?r* therapy"))) AND ((MeSH: (minors)) OR (Any Field: (minors)) OR (Any Field: (minor)) OR (MeSH: (child)) OR (Any Field: (child*)) OR (Any Field: (boy) OR Any Field: (boys)) OR (Any Field: (girl) OR Any Field: (girls)) OR (Any Field: (schoolchild)) OR (Any Field: (juvenil*)) OR (MeSH: (adolescent)) OR (Any Field: (adolescen*)) OR (Any Field: (youth*)) OR (Any Field: (teen*)) OR (Any Field: (pubescen*)) OR (MeSH: (pediatrics)) OR (Any Field: (p?ediatric*))) AND ((MeSH: (anxiety)) OR (Any Field: (anxiety)) OR (MeSH: (phobic disorders)) OR (Any Field: (phobi*)) OR (MeSH: (panic)) OR (Any Field: (panic)) OR (MeSH: (agoraphobia)) OR (Any Field: (agoraphobia)) OR (Any Field: (selective mutism)) OR (Any Field: (fear)) OR (Any Field: (worry))) AND ((AnyField:(meta-analysis)) OR (AnyField:("systematic review")) OR AnyField:(review* AND ("randomized controlled trial* OR "randomised controlled trial*" OR RCTs OR RCT)))

EMBASE

Session Results

.......................................................

No. Query Results

#60. #9 AND #36 AND #58 AND #59

#59. #54 OR #55 OR #56 OR #57

#58. #37 OR #38 OR #39 OR #40 OR #41 OR #42 OR #43 OR

#44 OR #45 OR #46 OR #47 OR #48 OR #49 OR #50 OR

#51 OR #52 OR #53

#57. review*:ti,ab AND ('randomized controlled

trials':ti,ab OR 'randomised controlled

trials':ti,ab)

#56. 'systematic review':ti,ab

#55. 'meta analysis'/de OR 'systematic review'/de

#54. 'meta analysis':ti,ab

#53. 'worry'/exp OR worry

#52. 'worrying'/exp OR worrying

#51. worries

#50. worried

#49. 'fear'/exp OR fear

#48. 'selective mutism'/exp OR 'selective mutism' OR

(selective AND ('mutism'/exp OR mutism))

#47. 'selective mutism'/exp OR 'selective mutism'

#46. 'mutism'/exp OR mutism

#45. 'agoraphobia'/exp OR agoraphobia

#44. 'panic attack'/exp OR 'panic attack' OR

(('panic'/exp OR panic) AND attack)

#43. 'panic disorder'/exp OR 'panic disorder' OR

(('panic'/exp OR panic) AND ('disorder'/exp OR

disorder))

#42. 'panic'/exp OR panic

#41. 'phobic disorders'/exp OR 'phobic disorders' OR

(phobic AND ('disorders'/exp OR disorders))

#40. 'phobia'/exp OR phobia

#39. 'anxiety disorder'/exp OR 'anxiety disorder' OR

(('anxiety'/exp OR anxiety) AND ('disorder'/exp

OR disorder))

#38. 'anxiety disorder'/exp OR 'anxiety disorder'

#37. 'anxiety'/exp OR anxiety

#36. #10 OR #11 OR #12 OR #13 OR #14 OR #15 OR #16 OR

#17 OR #18 OR #19 OR #20 OR #21 OR #22 OR #23 OR

#24 OR #25 OR #26 OR #27 OR #28 OR #29 OR #30 OR

#31 OR #32 OR #33 OR #34 OR #35

#35. school*:ti,ab

#34. paediatric*

#33. pediatric*

#32. 'pediatrics'/exp OR pediatrics

#31. pubescen*

#30. 'under age*'

#29. teen*

#28. youth*

#27. juvenil*

#26. 'juvenile'/exp OR juvenile

#25. adolescen*

#24. 'adolescent'/exp OR adolescent

#23. 'school child*'

#22. 'school child'/exp OR 'school child'

#21. schoolchild*

#20. child*

#19. 'child'/exp OR child

#18. kids

#17. kid

#16. girl*

#15. 'girl'/exp OR girl

#14. boyhood

#13. boys

#12. 'boy'/exp OR boy

#11. 'minors'/exp OR minors

#10. 'minor (person)'/exp OR 'minor (person)'

#9. #1 OR #2 OR #3 OR #4 OR #5 OR #6 OR #7 OR #8

#8. cognitive AND therapy:ti,ab

#7. behavio* AND therapy:ti,ab

#6. 'cognitive behavioural therapy'/exp OR 'cognitive

behavioural therapy' OR (cognitive AND

behavioural AND ('therapy'/exp OR therapy))

#5. 'cognitive behavioral therapy'/exp OR 'cognitive

behavioral therapy' OR (cognitive AND behavioral

AND ('therapy'/exp OR therapy))

#4. cbt

#3. 'cognitive behaviour therapy'/exp OR 'cognitive

behaviour therapy' OR (cognitive AND

('behaviour'/exp OR behaviour) AND ('therapy'/exp

OR therapy))

#2. 'cognitive behaviour therapy'/exp OR 'cognitive

behaviour therapy'

#1. 'cognitive behavioral therapy'/exp OR 'cognitive

behavioral therapy'

.......................................................
