# Supplementary material for: Do Parents Enhance Cognitive Behavior Therapy for Youth Anxiety? An Overview of Systematic Reviews Over Time
Source: Clin Child Fam Psychol Rev. 2023 May 22;26(3):773–88. doi: 10.1007/s10567-023-00436-5 (PMC10465628; doi:10.1007/s10567-023-00436-5)
Supplement: Supplementary file 3 — Supplementary file3 (DOCX 43 KB) [file 10567_2023_436_MOESM3_ESM.docx]

**Appendix C**

*Inclusion and exclusion criteria identified by reviews in overvies*

| Author (year) | Age range | Unpublished Studies | OCD  included | Subclinical  included | Non-RCTs included | Inclusion criteria | Exclusion criteria |
| --- | --- | --- | --- | --- | --- | --- | --- |
| Barmish & Kendall (2005) | 7-18 |  |  |  | X | - Written in English. - F-CBT assessed. - F-CBT minimum 4 parent sessions. | - Medication trials. - Non-CBT studies. - Primary diagnoses of SP and OCD. |
| James et al. (2005) | 6-18 | X |  |  |  | - RCTs of CBT vs. waitlist/attention control. - One or more diagnosed anxiety disorders. - Manualised CBT with minimum 8 sessions by trained therapist. | - SP, selective mutism, OCD and PTSD excluded. - No concurrent medication. - Endpoint data available for < 60% of sample. |
| In-Albon & Schneider (2007) | 6-18 |  |  |  |  | - RCT with treatment for anxiety vs. a control. - Written in English and German. - Primary anxiety disorder. - Standard treatment protocol. - Studies included *M*, *SD* and *N* at each time point. | - PTSD and OCD. - Treatment groups where *N* < 10. - Single case designs. - Subclinical cases.   Psychopharmacology studies. |
| Ishikawa et al. (2007) | 4-17 |  | X |  |  | - RCTs. - Treatment elements of CBT. - Written in English. - Trials that detailed statistics required for meta-analysis. - OCD and PTSD included. |  |
| Creswell & Cartwright-Hatton (2007) | 6-18 |  |  | X | X | - RCTs that compared F-CBT vs. Y-CBT for youth anxiety and F-CBT studies that were not controlled. - Primary anxiety disorder or severely anxious. | - Trials that exclusively treated OCD, PTSD or Simple Phobia. |
| Silverman et al. (2008) | Not  reported |  |  | X |  | - Treatments that targeted most prevalent phobic and anxiety disorders. - Studies needed to adhere to most methodological features per Chambless & Hollon (1998). - Group design studies which evaluated a combination of therapeutic strategies. | - Single case design series. - OCD or PTSD. |
| Fjermestad et al. (2009) | 6-18 | X |  |  |  | - RCTs. - CBT for youth anxiety.   ≥ 1 relationship factors associated with treatment outcome. | - OCD or PTSD |
| Reynolds et al. (2012) | < 19 |  | X | X |  | - RCTs - Studies published in English - Participants needed elevated anxiety, or formal anxiety diagnosis. - PTSD, OCD and SP included. - Received anxiety treatment.   *M* and *SD* reported. |  |
| Breinholst et al. (2012) | 6-18 |  |  |  |  | - RCTs. - Compared Y-CBT vs. F-CBT regardless of format. - Youth with primary anxiety disorder. |  |
| Thulin et al. (2014) | < 18 |  | X |  |  | - RCTs. - Published trials written in English. - Direct comparison between F-CBT vs. Y-CBT. - Diagnosed anxiety disorder using an evidence-based instrument. - OCD and PTSD included. - Primary anxiety disorders. |  |
| Manassis et al (2014) | 6-18 |  |  |  |  | - RCTs comparing CBT vs. waitlist or attention control. - Diagnosis of an anxiety disorder.   Outcome measures. | - OCD and PTSD. |
| James et al. (2015) | 4-18 | X |  |  |  | - RCTs (cross-over trials/cluster-randomised included). - Primary anxiety disorder. - Manualized and modular CBT. - Minimum nine sessions of direct youth contact. - Follow-up data with comparators. | - PTSD, OCD, SP and selective mutism. |
| Higa-McMillan et al. (2016) | 1-19 |  |  | X |  | - Random assignment. - Active psychosocial or combined treatment vs. control.   Anxiety-related outcome measures reported at post. | - Primarily OCD and PTSD. |
| Öst & Ollendick (2017) | ≤ 18 | X | X |  |  | - Random allocation to treatment or control. - Participants diagnosed with anxiety. - Published in English. - Received a brief or concentrated condition. | - Anxiety disorder is not principal. - Single case studies. - Non-RCTs. - Non-CBT. - Secondary analysis. |
| Zhang et al. (2017) | Mean  = < 7 |  |  |  |  | - RCTs. - Primary diagnosis of anxiety. - Any psychological treatment vs. control (waitlist and usual treatments). - Psychiatric comorbidity acceptable. | - 20% sample had primary diagnosis of PTSD and/or OCD. - Psychotherapies used in combination/augmentation/maintenance/relapse prevention - Non-anxiety specific treatment. - Drug placebo control. - Concomitant use of psychotropics. - Treatment duration < 6 weeks or number of sessions < 6. - *N* < 10. |
| Carnes et al. (2018) | Mean  = 7-13 | X |  | X | X | - Peer reviewed RCTs or quasi-experimental. - CBT for an anxious child and both parents. - Provided anxiety outcome data. - Subclinical cases. | - Youth with primary diagnosis of another mental health disorder, neurodevelopmental disorder or medical condition. |
| Zhou et al. (2019) | ≤ 18 | X |  |  |  | - RCTs. - No language restriction. - Any structured therapy. - Can be delivered in any modality. - Any control condition. - Primary diagnosis of anxiety. | - Combination therapies. - Treatment-resistant anxiety. - Treatment duration < 6 weeks - *N* < 10. - OCD and PTSD. |
| Comer et al. (2019) | Mean =  < 7.9 |  | X | X | X | - Includes non-randomized designs. - Treatment focused on anxiety-related problems. - Multiple baseline trials. - OCD and PTSD included. - Included subclinical designs. | - Case studies and case series with *N* < 5. |
| James et al. (2020) | < 19 | X |  |  |  | - RCTs, cross over trials and cluster randomized trials with non-CBT comparator. - Diagnosed anxiety disorder. - Permissible comorbidities. - All settings. - CBT, alone or in combination with medication. - Y-CBT, F-CBT and P-CBT. | - Samples only inclusive of PTSD and/or OCD. - CBT delivered online via digital devices. |
| Cardy et al. (2020) | 11-18 |  |  |  | X | - Includes non-randomized studies. - Published in English. - Met diagnosis ≥ 1 anxiety disorder. - Adolescent receives CBT as primary therapy. - Adolescent included in therapy. - Format of therapy could vary. - Minimum 1 biological parent involved. - Outcome data provided. - Participants could be taking psychotropic medication. | - PTSD and OCD. - Youth whose anxiety was secondary to physical health condition/neurodevelopmental disorder/learning disability/social impairment. - Studies that included or focused solely on foster parents, carers, guardians. |
| Sigurvinsdóttir et al. (2020) | $\leq$18 | X |  |  |  | - RCTs and cluster-randomised designs. - Primary diagnosis of anxiety. - CBT delivered alone/in combination with medication. - Reported youth anxiety outcomes including remission. - CBT of any format. | - Trauma, SP, selective mutism, OCD. |
| Guo et al. (2021) | < 18 | X |  |  |  | - RCTs, crossover trials and cluster randomised trials. - Primary diagnosis of anxiety - Compared individual CBT vs. group CBT in various formats. | - Treatment duration < 6 weeks/number of sessions < 6. |
| Peris et al. (2021) | 6-18 |  |  |  |  | - Principle diagnosis of primary anxiety disorder - RCT comparing F-CBT vs. Y-CBT. - CBT that focused on youth anxiety. - Youth participated in treatment. - Anxiety outcomes collected at pre-post. - Parenting/family outcomes collected at pre-post. - F-CBT defined as 1 family session or explicit inclusion of parenting skills for anxiety management, in addition to Y-CBT. | - Non-anxiety outcomes. - Studies where quantitative data not specified. - Group treatments. - Single-session CBT. |
| Yin et al. (2021) | ≤ 18 |  |  |  |  | - RCTs of P-CBT vs. waitlist or CBT with parents. - Primary diagnosis of anxiety disorder. - Psychiatric comorbidity acceptable. | - > 20% primary diagnosis of other mental disorder. - > 20% of took psychotropics for anxiety. - Treatment < 6 weeks/number of sessions < 6. |
| Jewell et al. (2022) | 4-17 | X |  |  | X | - Mothers and/or fathers of youths < 18. - Parents receive direct treatment. - Minimum 1 diagnosed anxiety disorder. - Comparator conditions. - Outcomes documented in interview.   Any quantitative design. | - Youth were direct participant. - Treatment included parent component as part of youth-focused intervention. - Non-anxiety treatments - Preventative interventions. - Interventions aimed at reducing parental stress. - PTSD/OCD/health anxiety. - Grandparents/teachers/other professionals as main participants. - Children ‘at risk’/subclinical.. - Specific groups (e.g., physical health problems, intellectual disability). - Focus on parent outcomes. - Qualitative methodology. |

*Note.* X = meets inclusion criteria for systematic review. SAD = separation anxiety disorder; SP = specific phobia; SoP = social phobia; GAD = generalised anxiety disorder; OCD = obsessive-compulsive disorder; PTSD = post-traumatic stress disorder; SAD = separation anxiety disorder.
